# A 2D laser rangefinder scans dataset of standard EUR pallets

**DOI:** 10.1016/j.dib.2019.103837

**Published:** 2019-03-22

**Authors:** Ihab S. Mohamed, Alessio Capitanelli, Fulvio Mastrogiovanni, Stefano Rovetta, Renato Zaccaria

**Affiliations:** a INRIA Sophia Antipolis - Méditerranée, Université Côte d’Azur, France; b Department of Informatics, Bioengineering, Robotics and Systems Engineering (DIBRIS), University of Genoa, Italy; c Teseo S.r.l., Genova, Italy

**Keywords:** 2D laser rangefinder, Object detection, Robotics, Automated guided vehicle

## Abstract

In the past few years, the technology of automated guided vehicles (AGVs) has notably advanced. In particular, in the context of factory and warehouse automation, different approaches have been presented for detecting and localizing pallets inside warehouses and shop-floor environments. In a related research paper Mohamed et al., 2018, we show that an AGVs can detect, localize, and track pallets using machine learning techniques based only on the data of an on-board 2D laser rangefinder. Such sensor is very common in industrial scenarios due to its simplicity and robustness, but it can only provide a limited amount of data. Therefore, it has been neglected in the past in favor of more complex solutions. In this paper, we release to the community the data we collected in Ref. Mohamed et al., 2018 for further research activities in the field of pallet localization and tracking. The dataset comprises a collection of 565 2D scans from real-world environments, which are divided into 340 samples where pallets are present, and 225 samples where they are not. The data have been manually labelled and are provided in different formats.

**Table:** Specifications Table

Subject area	Engineering
More specific subject area	Robotics, Object Detection, Automated Guided Vehicle
Type of data	Raw depth data provided by the 2D range sensor
	Processed 2D bitmap-like image representation of raw data
How data was acquired	2D laser rangefinder (SICK S3000 Pro CMS)
Data format	Files in text format *.txt*
	2D images in *.jpg*&*.png* (681×533 & 250×250*pixels*)
	MAT-files in MATLAB format *.mat*
Experimental factors	2D depth data processed offline and converted into 2D images.
	Images have been manually tagged whether they include a pallet
	or not, and eventually paired with the respective region of interest.
Experimental features	Raw data have been acquired by moving a 2D laser scanner
	in a realistic reproduction of a factory workshop, featuring
	pallets, people, robots and other equipment.
Data source location	EMARO Lab, Department of Informatics, Bioengineering, Robotics
	and Systems Engineering, University of Genoa, Genoa, Italy
	(44.402241, 8.960811)
Data accessibility	Dataset and codes are archived in a GitHub repository at:
	https://github.com/EmaroLab/PDT
Related research article	“Detection, localization and tracking of pallets using
	learning techniques and 2D range data” [1]

**Table:** 

**Value of the data** • The 2D Laser Rangefinder dataset allows to develop novel techniques for pallet detection, localization and tracking. • The 2D Laser Rangefinder dataset can be used as banchmark to compare the accuracy of different pallet detection, localization and tracking algorithms. • The 2D Laser Rangefinder dataset allows to improve Automated Guidance Vehicles (AGVs) in industrial workshop environments. • The 2D Laser Rangefinder dataset can be used to simulate the 2D range sensor data of a mobile robot moving in an industrial workshop environment. • To our knowledge, this is the first dataset for pallet localization and tracking using only 2D Laser Rangefinder data, as opposed to previous datasets aimed at generic AGV and/or more complex sensors [2], [3], [4], [5].

## 1 Data

In this article, we present a dataset of 2D range data obtained from a laser scanner moving inside an industrial workshop environment, where EUR standard pallets (see Fig. 3(a)), people, robots and other equipment are present. Each frame of the sensor trajectory corresponds to: (i) a 2D range scan (see Table 1) obtained from a SICK S3000 Pro CMS laser rangefinder (see Fig. 3(b)); (ii) a 2D image obtained by processing the 2D range scan (see Fig. 4); (iii) a tag attached by a human, indicating whether the scan includes a pallet or not; and (iv), the region of interest of the pallet in the image (if any), also defined by a human.

**Fig. 1:**
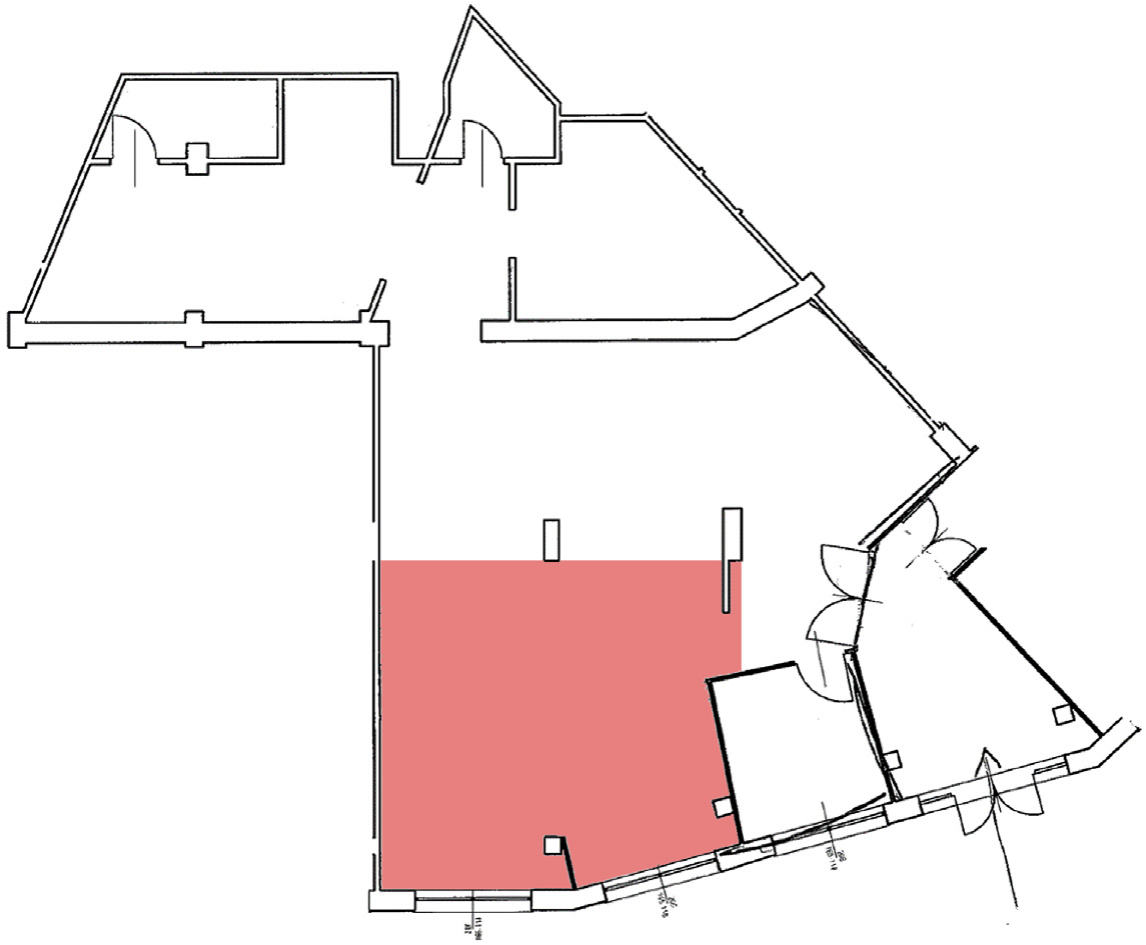
A planimetry of the indoor environment where the experiment took place. The 2D laser rangefinder has been moved along several trajectories inside the read area, measuring 40 m^2^. The rest of the environments is still visible in several frames. In the whole environments several pieces of furniture and equipment, pallets, robots as well as people were present.

**Fig. 2:**
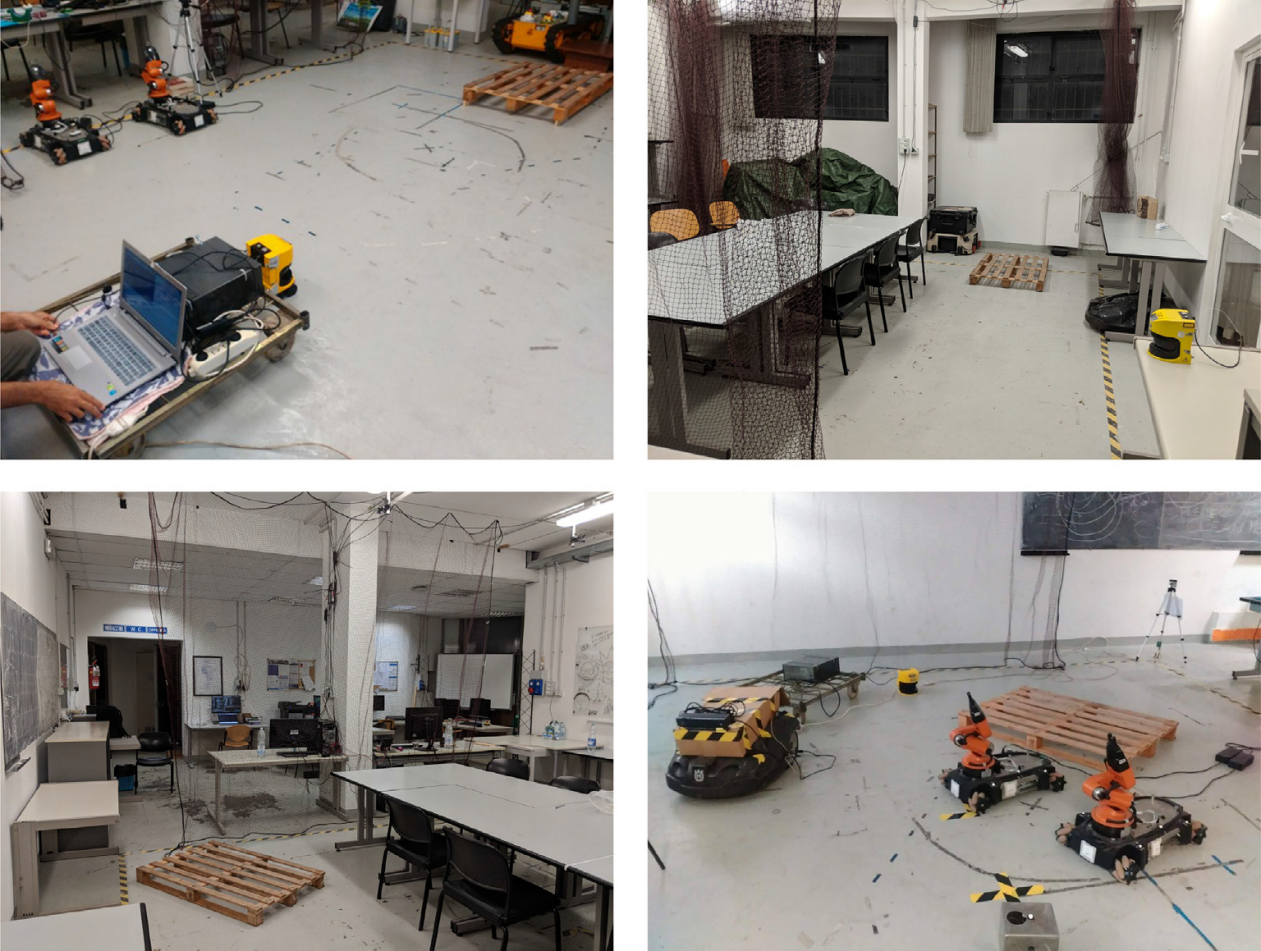
Snapshots of the test environment in different configurations. In the images, a number of other objects appear beyond pallets, such as other robots, equipment and furniture.

**Fig. 3:**
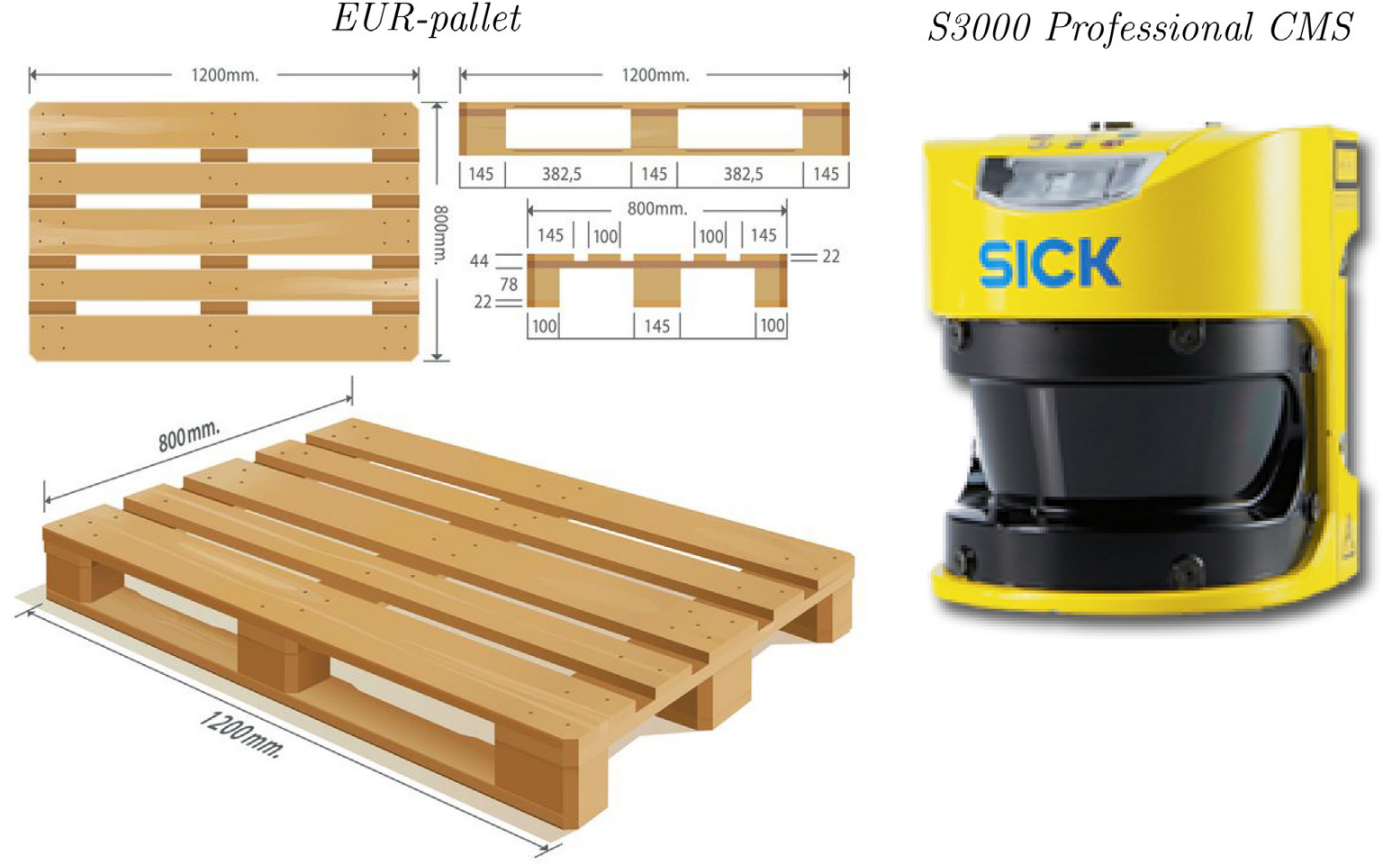
The equipment that has been used to acquire the raw 2D range data: on the left-hand side, the geometric characteristics of standard European pallet are shown, whilst on the right-hand side the S3000 Professional laser scanner (Type: S30A-6011DB) is represented.

**Table 1:**
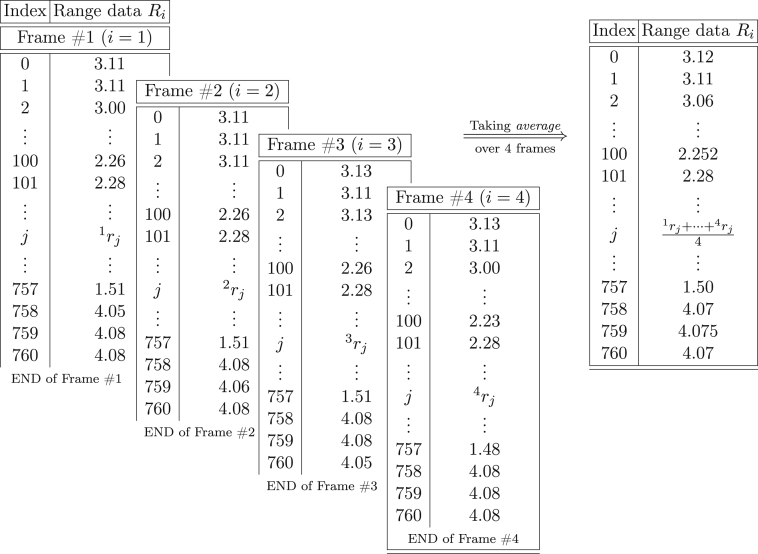
An example of the raw range data provided by the laser rangefinder. As soon as the data is visualized using the standard ROS package *rviz*, four sequential frames are stored in a text file. Then, the *average* can be calculated in order to perform the detection and tracking of the pallet using machine learning techniques.

**Fig. 4:**
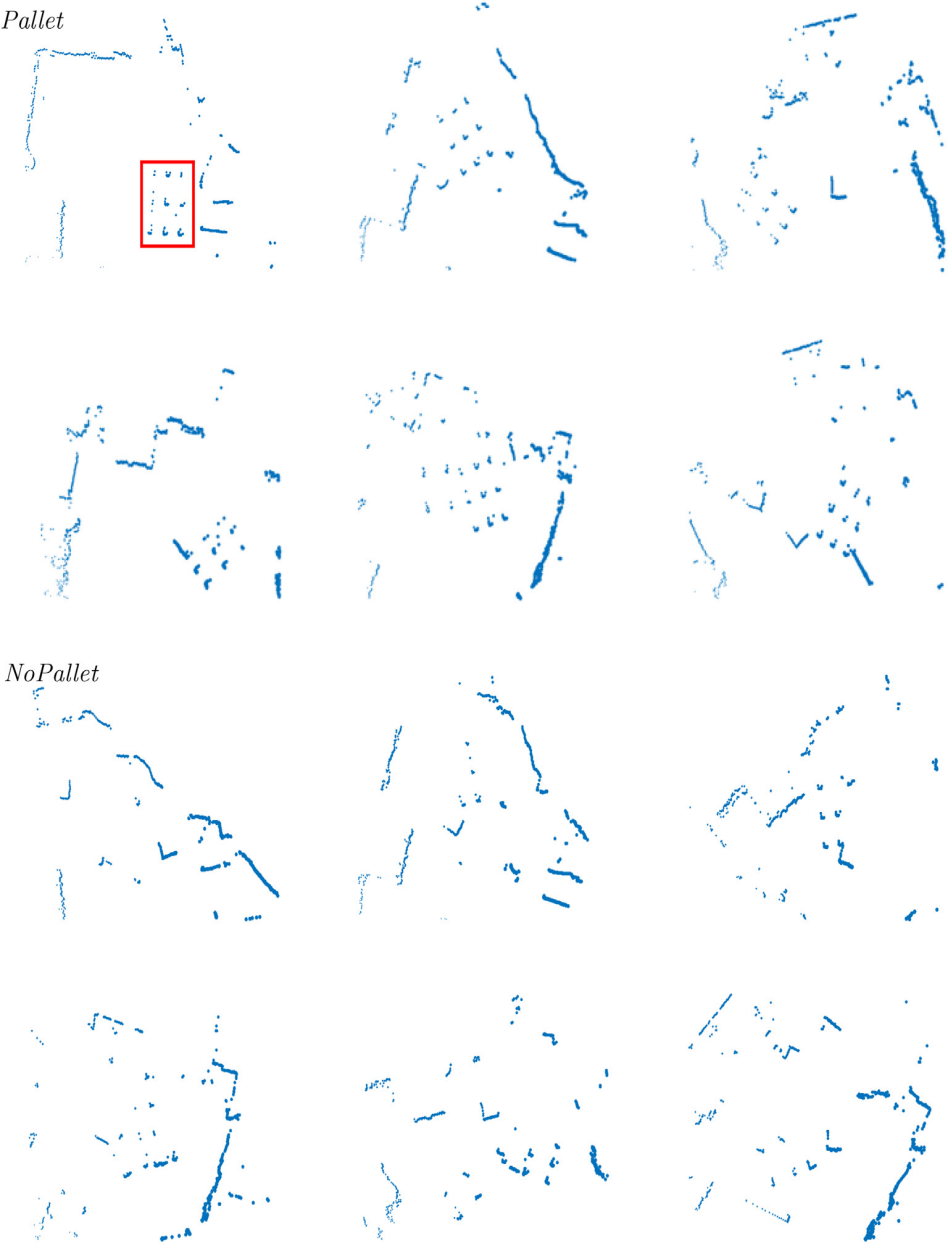
The dataset of real-world 2D scans represented in Cartesian coordinates: the first two rows are related to the case where a pallet is present in the environment and the operating face is free, whilst the last two rows represent samples of the dataset when no pallet is present or the operating face is not accessible by an autonomous forklift. The red box in the first image represents an example of region of interest, i.e., the part of the image actually where the pallet is located.

The laser rangefinder has a resolution of 0.25 deg and a maximum field of view of 190 deg, leading to scans made of 761 ranges. It operates at 16 Hz frequency, and the scans are averaged every 4 frames during the static data acquisition phase in order to reduce noise. There are a total of 565 scans, 340 of which contains a pallet, while the remaining 225 do not. The corresponding 2D images are obtained by converting the range data from polar to cartesian coordinates and resizing them to 250×250 px. Also, images containing a pallet come with a pallet Region Of Interest (ROI), defined by its upper-left and lower-right vertices. Finally, an additional set of 4 continuous trajectories’ raw range data is also made available, to allow online testing.

## 2 Experimental design, materials, and methods

### 2.1 Equipment and software

In our experiment, the data have been acquired using a commercial 2D laser rangefinder from SICK, in particular, the model S3000 Pro CMS^1^ pictured in Fig. 3(b). The sensor has a maximum range of 49 m (20 m at 20% reflectivity), a resolution of 0.25 deg, a 16 Hz refresh frequency, and an empirical error of 30 mm. The maximum field of view of the rangefinder is 190 deg, which is sufficient for the detection of objects in front of an eventual AGV. The sensor generates an array of 761 distances in polar coordinates, i.e., each value in the array correspond to the distance to the closest object for every angle in 0.25 deg increments.

The choice of this sensor was due to its widespread adoption in industrial mobile robotics, where it is mostly employed for safety applications and is appreciated for its robustness and precision. It belongs to the class of sensors based on the Time-of-Flight (TOF) principle, i.e., sensors which measure the delay between the emission of a signal and the moment it hits back a receiver in order to estimate the distance to a surface. This category of systems involves sensing devices such as Laser Measurement Systems (LMS) or LIDARs, radars, TOF cameras, and sonar sensors, and they emit either light or sound waves in the environment. Knowing the speed with which the signal propagates and using precise circuitry to measure the exact TOF, the distance can be estimated with high precision.

The laser rangefinder is then connected to a PC through a RS422-USB converter, which has a transmission rate of 500 kBaud. The PC used to acquire the data is equipped with an Intel^®^ Core i5-4210U 1.70 GHz CPU and 6 GB of RAM, and runs Ubuntu 16.04 64 bit.

On the software side, real-world data is acquired online using an *ad hoc* software^2^ running in the Robot Operating System framework^3^ (ROS). Offline processing (i.e., conversion to 2D images and manual definition of the regions of interest) has been performed in MATLAB. The scripts employed to that purpose and the resulting *.mat* files are also provided as part of this dataset.

### 2.2 Environment

We performed our experiments for data acquisition in the indoor environment represented in Fig. 1, Fig. 2, with the sensor moving in the 40 m^2^ area highlighted in Fig. 1. Such environment has been fitted to reproduce a typical industrial workshop, featuring industrial pallets, furniture, robots and equipment (e.g., a conveyor belt). People were also included in the scene and allowed to move during data acquisition, which lead to temporary occlusions of the objects in the environment. Between acquisition sessions, the position of several objects was modified to better simulate a dynamic environment. The 2D laser rangefinder was positioned close to the floor, in a way that was both realistic with real-world mounting position and able to perceive a pallet laying directly on the ground.

Concerning the type of pallet, we focused on the EUR-pallet standard depicted in Fig. 3(a), which is the European pallet format specified by the European Pallet Association (EPAL).^4^ The size of EUR-pallets is 1200 mm×800 mm with a height of 144 mm. Moreover, we defined as operating face of the pallet the one of narrower width. On that face, there are two slots, each 227.5 mm wide.

### 2.3 Experiments

In our experiments, the sensor was moved around the environment. Sensor frames differ from each other by the position and orientation of the pallet with respect of the sensor, but also due to the dynamic nature of the environment, as described in the previous section. In particular, it is possible that the pallet is heavily occluded and only few points belonging to it are visible in the frame.

The acquired raw range data $R_{i}$ at any time instant *i* represent the array of measured distances from the rangefinder to surrounding objects in the environment in the direction given by the angle $\phi_{j}$. More formally:

(1) $$R_{i}=\left\{r_{0},\ldots ,r_{j},\ldots ,r_{M}\right\},$$

where *M* is the maximum number of range points acquired per frame, which is related to the sensor's field of view and angular resolution. In our case, M=761, as the two values are 190 deg and 0.25 deg respectively. Keep in mind that the sensor employed runs at 16 Hz, which would rapidly lead to an unmanageable amount of data, especially considering the manual labelling steps ahead. For this reason, we decided to effectively reduce the operating frequency to 4 Hz in the static data acquisition phase, thus every $R_{i}$ is actually the result of the average of 4 raw consecutive frames from the sensor. This also helps reducing noise on the data. An example of such process as well and the structure of the raw range data are reported in Table 1.

In our experiments, we are focusing on the detection of pallets in the environment, hence, the set *R* of all raw range data readings $R_{i}$, consisting of 565 2D range scans, has been manually divided into two classes:

1. *Pallet* class represents the case of having a pallet located somewhere in the environment with a free operating face, i.e., it can be eventually be picked up by an AGV as an autonomous forklift. It consists of 340 samples.
2. *NoPallet* class represents the case in which no pallet is present in the environment, or there is, but the operating face is too cluttered to allow an AGV such as an autonomous forklift to pick up the pallet. It consists of 225 samples.

This manual labeling step has been performed with the help of an online ROS visualization tool, *RViz*.^5^ An operator checked the screen of the PC while the sensor was being moved, marking frames where a pallet with a free operating face was present in the sensor's FOV.

Afterwards, any range data frame $R_{i}$ can also be represented as a set $S_{i}$ of polar coordinates, and consequently converted to Cartesian coordinates using (2) and (3).

(2) $$s_{i}=\left\{\left(r_{0},\phi_{0}\right),\ldots ,\left(r_{j},\phi_{j}\right),\ldots ,\left(r_{M-1},\phi_{M-1}\right)\right\}.$$

(3) $$\{\begin{array}{c} x_{j}=r_{j}\;\cos\left(\phi_{j}\right),\\ y_{j}=r_{j}\;\sin\left(\phi_{j}\right). \end{array}$$

This results in a binary 2D image of the operating area's floor plan, which is then resized to 250×250 px. An example of the resulting images is given in Fig. 4.

Of course, these images are labelled with the same class as the originating frame. In particular, images belonging to the *Pallet* class come with the respective pallet ROI expressed as its upper-left and lower-right vertices (i.e., ($x_{min},y_{min}$) and ($x_{max},y_{max}$)), as well as a companion 250×250 px image containing the pallet only. Such ROIs are the results of the Region Proposal Network we employed in the related research paper [1]. The resulting ROIs have been manually labelled to indicate whether they present a pallet or another object. A selection of ROIs not including a pallet is also included in the dataset repository.

We will not further delve here into the details of our specific solution to the problem of pallet localization and tracking, which we present instead in the related research paper [1]. We just point out that the data was indeed employed for pallet localization and tracking and that the proposed architecture was tested using 4 additional continuous trajectories, which are also made available on the dataset repository. In particular, localization was performed using the aforementioned Region Proposal Network, cascaded with a Faster Recurrent Convolutional Neural Network classifier that took as input the set of manually labelled ROIs [6]. On the other hand, tracking was performed using a Kalman Filter [7]. The filter was also used to implement a Sequential Classification procedure, i.e., accepting a ROI as an actual pallet was deferred till it was detected and tracked for a predefined amount of time, eventually reaching a sufficient confidence threshold.

Finally, note that the dataset can be used for multi-pallet detection, but that was not part of our data collection experiment. Indeed, in the related research paper [1] we ran a preliminary study on the subject by generating artificial data. We want to stress that given that the EUR-Pallet is an official standard with strict tolerances, differences between any two pallets are not perceivable by the sensor, due to its characteristics and margin of error. This leads to two major consequences:

• It is not possible with this sensor and with the dataset we provide to univocally identify a pallet, yet it is possible to distinguish them from each other if appropriate tracking techniques are put in place, as we did in the related research paper [1].
• Artificial 2D images including two or more pallets in every image are easy to generate. This can be achieved by taking an original image and adding the pallet ROI from another image, possibly changing position, orientation, and/or adding noise, and consequently deleting any reading in the original image that would now be occluded by the new pallet. Such artificial images are not provided here, but can easily be generated with the provided materials and tools. Nevertheless, future work on our related research paper will include real-world multi-pallet testing, thus an extended dataset will be made available too.

### 2.4 Dataset inspection

The dataset is completely contained in the *AllData* folder of the provided git repository. The folder is structured as follows:

• The *Class1* and *Class2* folders correspond to *Pallet* and *NoPallet* classes, respectively. They include 565 raw laser rangefinder scans in *.txt* format in total, 340 for the former class and 225 for the latter.
• *DataSet565.mat* is a file containing the whole dataset as a 761×565 MATLAB matrix.
• *PalletImages* folder containing all the 250×250 px images in various formats, divided by class and eventually accompanied by the relative pallet's ROI. In particular, the files *PalletGrayImages.zip* and *RGBImages.tar.gz* contains the images in *.jpg* and *.png* format, respectively.
• *TrajectoryDataset* folder contains 4 additional continuous trajectories that we used to test the architecture presented in our related research paper [1]. The trajectories are provided in *.mat* format.

## Footnotes

1 https://www.sick.com/ag/en/s3000-professional-cms-sensor-head-with-io-module/s30a-6011db/p/p31284.

2 https://github.com/RobotnikAutomation/s3000_laser.

3 http://www.ros.org/about-ros/.

4 https://en.wikipedia.org/wiki/EUR-pallet.

5 http://wiki.ros.org/rviz.
